# An echocardiographic study of right ventricular function and pulmonary systolic pressure in patients treated with anthracyclines

**DOI:** 10.1186/s44348-026-00069-6

**Published:** 2026-03-25

**Authors:** Ramazan Ozan, Deniz Elçik, Alparslan Demiray, İskan Zengin, Erlan Abibulaev, Rıdvan Yurt, Orhan Ulaş, İsmail Düzgün, Aysu Çiçekli Aslan, Mevlüde Inanç, Abdurrahman Oğuzhan

**Affiliations:** 1 Department of Cardiology, Ağrı Training and Research Hospital, Ağrı, Turkey; 2 Department of Cardiology, Faculty of Medicine, Erciyes University, Kayseri, Turkey; 3 Department of Internal Medicine, Kayseri City Hospital, Kayseri, Turkey; 4 Department of Cardiology, Kayseri City Hospital, Kayseri, Turkey; 5 Department of Cardiology, Memorial Hospital, Kayseri, Turkey; 6 Division of Oncology, Department of Internal Medicine, Faculty of Medicine, Erciyes University, Kayseri, Turkey

**Keywords:** Anthracycline-induced cardiotoxicity, Ventricular Dysfunction, Right, Tricuspid annular plane systolic excursion (TAPSE), Systolic pulmonary artery pressure (sPAP), TAPSE/sPAP ratio, Hypertension, Pulmonary

## Abstract

**Background:**

Anthracycline-based chemotherapy agents are widely used and are highly effective, particularly for breast cancer treatment. Although the cardiotoxic effects of anthracyclines on left ventricular (LV) function are well established, their impact on right ventricular (RV) function has not been sufficiently investigated. This study aimed to evaluate the effects of anthracycline therapy on RV function and to compare them with LV function to determine the potential cardiotoxic effects on both ventricles.

**Methods:**

This single-center retrospective cohort study included 38 female patients with breast cancer who were treated with anthracyclines between January 2021 and June 2023. Echocardiographic parameters and cardiac biomarkers were evaluated at baseline and at 6-month follow-up visit. LV ejection fraction (LVEF) was calculated using the Teichholz method due to the retrospective design. RV function was assessed by tricuspid annular plane systolic excursion (TAPSE), systolic pulmonary artery pressure (sPAP), and the TAPSE/sPAP ratio. Cancer therapy–related cardiac dysfunction (CTRCD) was defined according to current European Society of Cardiology criteria. Serum troponin I and pro–brain natriuretic peptide levels were recorded. Paired comparisons were performed using the paired-samples t-test.

**Results:**

Following anthracycline therapy, LV end-systolic diameter increased (2.76 ± 0.24 cm vs. 3.03 ± 0.29 cm, *P* < 0.001), and LVEF decreased (67.3% ± 3.6% vs. 62.2% ± 4.5%, *P* < 0.001). No patient fulfilled the guideline-defined criteria for CTRCD. Early diastolic transmitral flow velocity (E wave) and mitral annular early diastolic velocity (e′) were reduced (E: 0.63 ± 0.16 m/sec vs. 0.52 ± 0.12 m/sec, *P* < 0.001; e′: 0.09 ± 0.03 m/sec vs. 0.07 ± 0.02 m/sec, *P* = 0.001). TAPSE decreased (2.28 ± 0.36 cm vs. 2.16 ± 0.27 cm, *P* = 0.047), and systolic pulmonary artery pressure showed a nonsignificant upward trend after treatment (*P* = 0.052). The TAPSE/sPAP ratio declined (1.11 ± 0.47 vs. 0.86 ± 0.20, *P* < 0.001), and pulmonary artery diameter increased (19.9 ± 2.0 mm vs. 21.3 ± 2.6 mm, *P* = 0.008). Serum troponin I levels increased significantly (4.84 ± 1.25 ng/L vs. 11.93 ± 4.91 ng/L, *P* < 0.001).

**Conclusions:**

Anthracycline therapy may be associated with modest changes in both LV and RV parameters. Reductions in TAPSE and the TAPSE/sPAP ratio, together with a nonsignificant upward trend in systolic pulmonary artery pressure, may reflect subtle alterations in RV–pulmonary arterial interactions rather than overt RV dysfunction. Routine evaluation of RV function, alongside LV assessment, may provide additional insights during cardiotoxicity monitoring in anthracycline-treated patients. These findings should be interpreted cautiously and confirmed in larger prospective studies.

## Background

Breast cancer accounts for approximately 30% of newly diagnosed malignancies in women worldwide [1]. Treatment strategies vary depending on molecular subtype, tumor stage, and recurrence risk. The main therapeutic modalities include surgery, chemotherapy, targeted therapy, radiotherapy, and endocrine therapy [2].

Anthracyclines are antineoplastic agents widely used in the treatment of breast cancer and other malignancies, including endometrial, gastric, and small-cell lung cancers, as well as lymphomas and leukemias [3]. Despite its proven efficacy in improving survival, anthracycline-based chemotherapy is associated with major adverse effects such as cardiotoxicity. Cardiac injury may occur during treatment or manifest years later, and its development is typically dose-dependent and often irreversible [4, 5]. The cardiotoxic mechanism is primarily related to oxidative stress and topoisomerase II inhibition, leading to myocardial cell apoptosis and structural remodeling [6].

Because cardiotoxicity can develop long after chemotherapy completion, lifelong multidisciplinary follow-up involving both cardiology and oncology specialists is critical [7]. Most previous studies have focused on left ventricular (LV) systolic and diastolic function as key predictors of cardiotoxicity [8]. However, the potential impact of anthracyclines on right ventricular (RV) function has not been thoroughly investigated. In particular, the role of RV diastolic dysfunction in the prediction of cardiotoxicity remains unclear [9]. Therefore, this study aimed to evaluate the effects of anthracycline therapy on right ventricular function and compare them with LV changes to determine the potential cardiotoxic effects on both ventricles.

## Methods

### Ethics statement

This study was conducted in accordance with the principles of the Declaration of Helsinki. The study protocol was reviewed and approved by the Erciyes University Clinical Research Ethics Committee (No. 2023/450). Due to the retrospective design and use of deidentified data, the requirement for written informed consent was waived.

### Study design

This single-center retrospective cohort study was conducted between January 2021 and June 2023 to evaluate cancer therapy–related cardiac dysfunction (CTRCD) in patients receiving anthracycline-based chemotherapy for breast cancer. Owing to the exploratory nature of the study and its relatively limited sample size, the analyses were considered hypothesis-generating.

### Study population

A total of 38 female patients with breast cancer (mean age, 49 ± 9 years) who received anthracycline therapy and underwent regular echocardiographic follow-up at a single tertiary center were included. Patients with congestive heart failure, coronary artery disease, history of pulmonary thromboembolism, chronic obstructive pulmonary disease, asthma, arrhythmia, moderate or severe valvular heart disease, cardiac metastases, or thoracic radiotherapy were excluded to minimize potential confounding effects.

Demographic data (age, weight, and body surface area [BSA]) and comorbidities were recorded. BSA was calculated using the Mosteller formula:

$$\text{BSA }\left({\mathrm{m}}^{2}\right)=\sqrt{\frac{\text{Height }\left(\mathrm{cm}\right)\times \text{Weight }(\mathrm{kg})}{3600}}$$

The cumulative anthracycline dose ranged from 356 to 480 mg/m^2^ (mean, 435.2 ± 37.6 mg/m^2^). The coefficient of variation (8.65%) indicated a relatively homogeneous dose distribution. This dose range reflects the standard institutional chemotherapy protocols applied during the study period and is consistent with commonly used doxorubicin-equivalent regimens in routine breast cancer practice. Given the well-established dose-dependent nature of anthracycline cardiotoxicity, reporting the cumulative dose distribution was considered important for appropriate interpretation of the echocardiographic and biomarker findings.

CTRCD was defined according to current European Society of Cardiology (ESC) guidelines as a reduction in LV ejection fraction (LVEF) of ≥ 10 percentage points to a value < 50%.

### Echocardiographic assessment

Comprehensive transthoracic echocardiography was performed using a Vivid 7 system (GE Healthcare) at baseline and at 6-month follow-up. Standard parasternal long-axis, short-axis, and apical views were obtained according to American Society of Echocardiography (ASE) recommendations.

LV structure and function were assessed using LV end-diastolic diameter (LVEDD), LV end-systolic diameter (LVESD), interventricular septal thickness, posterior wall thickness, LVEF, and the LV Tei index. Due to the retrospective design and limited availability of archived apical images, LVEF was calculated using the Teichholz method, which may be less sensitive to subtle volumetric changes compared with the biplane Simpson method.

Right ventricular function was evaluated using RV fractional area change (RVFAC), systolic tricuspid annular velocity (RV S′), diastolic velocities (RV e′ and RV a′), tricuspid annular plane systolic excursion (TAPSE), systolic pulmonary artery pressure (sPAP), and the TAPSE/sPAP ratio as an index of RV–pulmonary artery coupling. All measurements were performed by an experienced cardiologist blinded to clinical and laboratory data. To ensure measurement reliability and reduce inter- and intra-observer variability, all echocardiographic examinations were performed by the same experienced cardiologist using standardized acquisition and measurement protocols in accordance with ASE recommendations [10].

### Biochemical analyses

Venous blood samples were collected from the antecubital vein in the morning (09:00–10:00 am) after a 12-h fasting period, both before the initiation of anthracycline therapy and at the 6-month follow-up visit. The samples were placed in tubes containing tripotassium ethylenediaminetetraacetic acid and analyzed on the same day. Serum troponin I and pro–brain natriuretic peptide (proBNP) levels were retrospectively obtained from the institutional electronic medical record system to assess biochemical evidence of cardiotoxicity. Due to the retrospective design, cardiac biomarker measurements were not systematically obtained according to a predefined research protocol. Troponin I levels were measured based on routine clinical practice, which resulted in incomplete data for some patients.

### Statistical analysis

Statistical analyses were conducted using IBM SPSS ver. 22.0 (IBM Corp). The distribution of continuous variables was tested using the Shapiro–Wilk test. Normally distributed data are expressed as mean ± standard deviation, and non-normally distributed data as median (interquartile range). Comparisons between pretreatment and posttreatment variables were made using the paired-samples t-test for normally distributed data and Wilcoxon signed rank test for non-normally distributed data. Categorical variables were compared using the chi-square test. Statistical significance was set at *P* < 0.05.

## Results

This study included 38 female patients with breast cancer who underwent anthracycline therapy (mean age, 49 ± 9 years). Most participants (78.9%) had no chronic comorbidities, while 13.2% had diabetes, and 7.9% had hypertension (Table 1). Mean BSA was 1.83 ± 0.18 m^2^. Baseline BSA values were relatively homogeneous across the cohort, suggesting that cardiac chamber measurements were not substantially influenced by body size differences. After anthracycline treatment, LVEDD showed a slight, nonsignificant increase (P = 0.054). LVESD increased significantly (*P* < 0.001), whereas interventricular septal thickness and posterior wall thickness did not change significantly (P = 0.058 and P = 0.639, respectively).

**Table 1:** Chronic disorders and demographic data of the patients receiving anthracyclines (n = 38)

Characteristic	Value
Chronic disorder	
No chronic disease	30 (78.9)
Diabetes mellitus	5 (13.2)
Hypertension	3 (7.9)
Age (yr)	49 ± 9
Body weight (kg)	74.9 ± 14.3
Body surface area (m^2^)	1.83 ± 0.18

Values are presented as number (%) or mean ± standard deviation

**Fig. 1:**
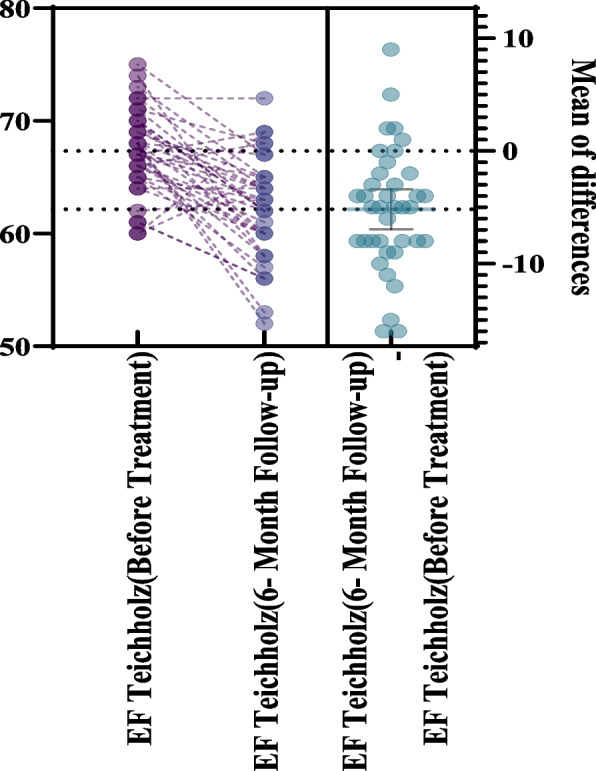
The effects of anthracycline therapy on left ventricular ejection fraction (EF)

Among diastolic parameters, early transmitral flow velocity (E) and mitral annular early diastolic velocity (e′) decreased significantly (*P* < 0.001 and P = 0.001, respectively), while the E/e′ ratio remained unchanged (P = 0.544). LVEF decreased from 67.3% ± 3.6% to 62.2% ± 4.5% (*P* < 0.001) but remained within normal range in most patients. According to the ESC guideline criteria, CTRCD, defined as a ≥ 10% absolute decline in LVEF to < 50%, was not observed in the study cohort. Therefore, the observed echocardiographic changes should be interpreted as subclinical functional alterations rather than guideline-defined cardiotoxicity. The LV Tei index remained stable, with no statistically significant difference between baseline and follow-up (baseline, 0.41 ± 0.12 vs. 0.40 ± 0.15; mean difference, − 0.012; 95% confidence interval, − 0.076 to 0.051; P = 0.694) (Table 2, Fig. 1).

**Table 2:** LV echocardiographic data of patients receiving anthracyclines (n = 38)

Echocardiographic parameter	Before treatment	After treatment^a^	*P*-value
LVEDD	4.41 ± 0.34	4.55 ± 0.40	0.054
LVESD	2.76 ± 0.24	3.03 ± 0.29	< 0.001
Interventricular septal thickness	0.96 ± 0.15	0.88 ± 0.17	0.058
Posterior wall thickness	0.91 ± 0.12	0.92 ± 0.13	0.639
LV E	0.63 ± 0.16	0.52 ± 0.12	< 0.001
LV e’	0.09 ± 0.03	0.07 ± 0.02	0.001
LV E/e’ ratio	7.2 ± 1.5	7.4 ± 2.2	0.544
LVEF^b^ (%)	67.3 ± 3.6	62.2 ± 4.5	< 0.001
LV Tei index^c^	0.41 ± 0.12	0.40 ± 0.15	0.694

Values are presented as mean ± standard deviation

LV, left ventricular; LVEDD, left ventricular end-diastolic diameter; LVESD, left ventricular end-systolic diameter

^a^Six-month follow-up echocardiography. ^b^Teichholz method. ^c^Myocardial performance index

**Fig. 2:**
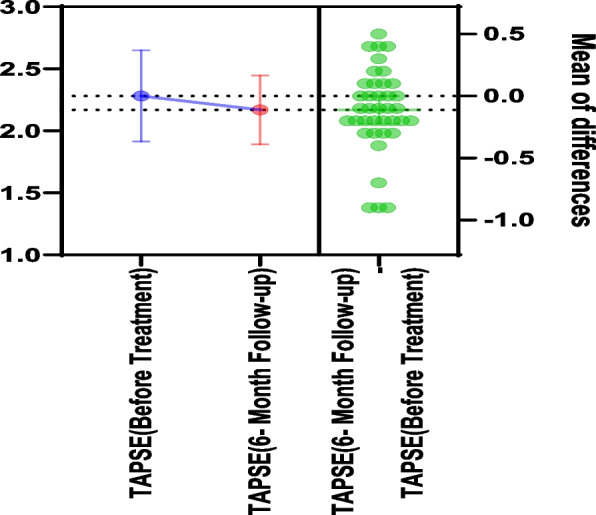
Tricuspid annular plane systolic excursion (TAPSE) values before and after treatment

**Fig. 3:**
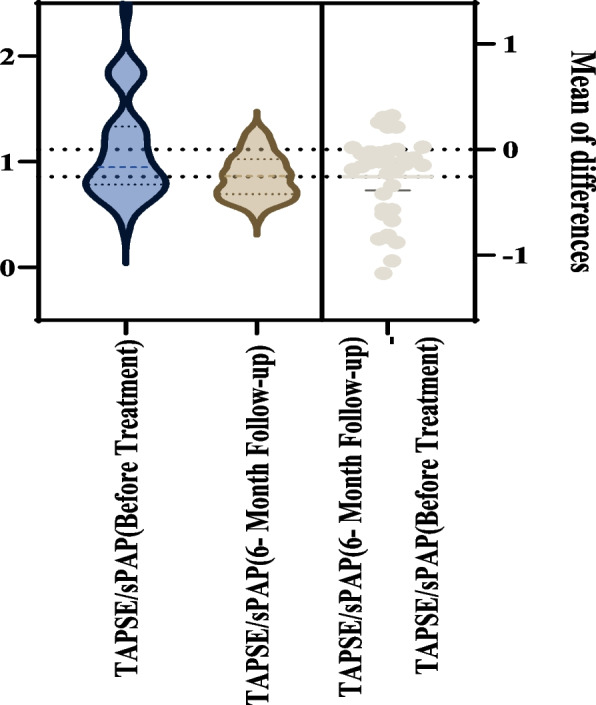
Alterations in tricuspid annular plane systolic excursion (TAPSE) to systolic pulmonary artery pressure (sPAP) ratio before and after treatment

At the 6-month follow-up, sPAP showed a borderline increase from 23.66 ± 8.86 to 26.34 ± 5.64 mmHg (*P* = 0.052). TAPSE decreased modestly (*P* = 0.047), and the TAPSE/sPAP ratio declined significantly from 1.11 ± 0.47 to 0.86 ± 0.20 (*P* < 0.001), suggesting a relative reduction in RV–pulmonary artery coupling (Figs. 2, 3). However, absolute values generally remained within reference ranges. Pulmonary artery diameter increased (*P* = 0.008). Additional changes were observed in RV A velocity (*P* < 0.001) and RV E/A ratio (*P* = 0.046) (Table 3).

**Table 3:** RV echocardiographic data of the patients receiving anthracyclines (*n* = 38)

Echocardiographic parameter	Before treatment	After treatment^a^	*P*-value
RVFAC	61.45 ± 10.1	60.6 ± 10.8	0.705
RV E (cm/sn)	0.46 ± 0.11	0.49 ± 0.12	0.138
RV e’(cm/sn)	0.11 ± 0.035	0.11 ± 0.034	0.961
RV A(cm/sn)	0.48 ± 0.10	0.61 ± 0.14	< 0.001
RV a’(cm/sn)	0.16 ± 0.039	0.16 ± 0.04	0.313
RV E/e’ ratio	4.32 ± 1.62	4.95 ± 2.26	0.085
RV E/A ratio	0.98 ± 0.35	0.86 ± 0.26	0.046
RV S′	11.95 ± 2	12.39 ± 2.15	0.315
Pulmonary artery diameter	19.89 ± 2	21.26 ± 2.61	0.008
Tricuspid regurgitation peak velocity (*n* = 36)	2.09 ± 0.49	2.28 ± 0.31	0.020
sPAP (mmHg) (*n* = 36)	23.66 ± 8.86	26.34 ± 5.64	0.052
TAPSE	2.28 ± 0.36	2.16 ± 0.27	0.047
IVC diameter (*n* = 37)	16.43 ± 2.8	15.3 ± 3.6	0.119
RV Tei index^b^	0.38 ± 0.15	0.41 ± 0.16	0.379
RV EDT (*n* = 36)	185.2 ± 37.2	169.5 ± 33.9	0.028
TAPSE/sPAP ratio (*n* = 36)	1.11 ± 0.47	0.86 ± 0.20	< 0.001

EDT, E-wave Deceleration Time; IVC, inferior vena cava; RV, right ventricular; RVFAC, right ventricular fractional area change; sPAP, systolic pulmonary artery pressure; TAPSE, tricuspid annular plane systolic excursion

^a^Six-month follow-up echocardiography. ^b^Myocardial performance index

No significant difference was observed in proBNP levels before and after treatment (*P* = 0.802). Troponin I levels were available in 21 patients (55% of the cohort) and showed a significant increase after anthracycline therapy (P < 0.001) (Table 4). Baseline demographic and echocardiographic characteristics did not significantly differ between patients with and without available troponin measurements (all *P* > 0.05), suggesting no evident selection bias. However, given the incomplete data and retrospective measurement strategy, these findings should be interpreted cautiously.

**Table 4:** Biochemical parameters of patients receiving anthracyclines

Biochemical parameter	Before treatment	After treatment^a^	*P*-value
ProBNP (pg/mL) (*n* = 37)	68.61 ± 42.49	67.01 ± 42.89	0.802
Troponin I (ng/mL) (*n* = 21)	4.84 ± 1.25	11.93 ± 4.91	< 0.001

ProBNP, pro–B-type natriuretic peptide

^a^Six-month follow-up echocardiography

## Discussion

This study evaluated the effects of anthracycline therapy on LV and RV function as well as cardiac biomarkers in breast cancer patients. The principal findings were modest reductions in LVEF and early diastolic velocities (E and e′), an increase in LVESD, and decreases in TAPSE and the TAPSE/sPAP ratio. These changes may reflect subclinical alterations in ventricular performance and RV–pulmonary arterial interaction rather than overt cardiac dysfunction. Despite stable N-terminal proBNP levels, a significant increase in serum troponin I was observed. The clinical relevance of these subtle but statistically significant changes warrants further clarification in larger prospective studies.

In agreement with previous reports, our study demonstrated reductions in LVEF and diastolic parameters after anthracycline therapy, suggesting early functional impairment. However, although the decline in LVEF (from 67.3% ± 3.6% to 62.2% ± 4.5%, P < 0.001) was statistically significant, values remained within the normal range and did not meet established cardiotoxicity thresholds. These findings are consistent with the 2023 ESC Cardio-Oncology Guidelines, which emphasize that subclinical myocardial dysfunction may occur even when LVEF remains preserved [11]. LVESD also increased significantly following treatment, supporting the presence of mild structural remodeling. These findings are consistent with earlier studies, which reported dose-related LV dysfunction and the potential benefits of cardioprotective therapy [12, 13].

LV diastolic abnormalities, including reduced E and e′ velocities, have also been reported in other studies [14–18]. The present results support the concept that diastolic impairment may precede overt systolic dysfunction and may represent an early manifestation of subclinical cardiotoxicity [19]. The increase in the LV Tei index observed in our cohort did not reach statistical significance, and therefore should be interpreted cautiously, although its upward trend is in line with previous data suggesting that the Tei index may reflect combined systolic–diastolic changes [20–24].

While most prior investigations focused primarily on LV performance, our findings suggest that RV parameters may also exhibit mild variation following anthracycline exposure. The observed reductions in TAPSE and the TAPSE/sPAP ratio together with a nonsignificant upward trend in systolic pulmonary artery pressure may reflect subtle changes in RV–afterload interaction rather than definite RV systolic dysfunction. Importantly, absolute values remained largely within reference ranges, and other established RV indices, including RVFAC and RV S′, did not demonstrate significant deterioration. Therefore, these findings should be interpreted as subclinical functional variations rather than overt RV impairment. These observations are consistent with limited prior reports suggesting that anthracyclines may affect both ventricles [25].

Other emerging evidence also indicates that anthracycline therapy can influence RV performance. A 2024 meta-analysis including more than 1,200 patients demonstrated reductions in RV systolic indices following anthracycline exposure [26], and a prospective cohort of 83 female patients reported early deterioration in RV global longitudinal strain after treatment [27]. Although the TAPSE/sPAP ratio has not been specifically validated in the context of anthracycline-induced cardiotoxicity, it is widely recognized as a surrogate of RV–pulmonary arterial coupling. Experimental and clinical studies by Guazzi et al. [26], Tello et al. [27], and Cameli et al. [28] all suggested that reductions in TAPSE/sPAP may indicate early RV–arterial uncoupling before the development of overt RV failure. Nevertheless, given the modest absolute changes observed in our cohort and the relatively small sample size, the clinical relevance of these findings remains uncertain and warrants confirmation in larger prospective studies.

Anthracycline-induced cardiotoxicity is thought to result from oxidative stress, topoisomerase II inhibition, mitochondrial injury, and apoptosis of cardiomyocytes [6, 11]. Furthermore, anthracycline cardiotoxicity is well known to be dose-dependent. In our cohort, the cumulative anthracycline dose was relatively homogeneous and consistent with standard clinical chemotherapy protocols, which reduces dose-related variability between patients but may also partly explain the observed subclinical functional changes. Therefore, the detected alterations in ventricular function and biomarkers should be interpreted within the context of cumulative dose exposure.

In our study, serum troponin I levels increased significantly after treatment, which may indicate biochemical evidence of myocardial stress. However, troponin measurements were only available in a subset of patients and were obtained according to routine clinical practice rather than a predefined study protocol. Although baseline characteristics did not differ between patients with and without available troponin data, the possibility of residual bias cannot be excluded. Therefore, these findings should be interpreted with caution. In contrast, proBNP levels remained unchanged, possibly reflecting the relatively early assessment period before overt neurohormonal activation occurs. The present findings reinforce the importance of comprehensive serial cardiac monitoring in patients receiving anthracyclines that incorporates both LV and RV parameters.

Several limitations of this study should be acknowledged. First, the retrospective design and the relatively small sample size from a single center may limit statistical power and generalizability; therefore, the findings should be interpreted as hypothesis-generating rather than definitive. In addition, advanced imaging modalities such as global longitudinal strain, three-dimensional echocardiography, and cardiac magnetic resonance were not available. Current cardio-oncology guidelines emphasize the importance of strain imaging for the early detection of subclinical cardiotoxicity, as deformation abnormalities may precede reductions in LVEF. The absence of strain analysis may therefore have limited the sensitivity of our study in identifying subtle myocardial dysfunction. In this context, parameters such as the TAPSE/sPAP ratio may represent a practical and widely available echocardiographic tool for the assessment of RV–pulmonary arterial interaction, particularly in retrospective or resource-limited settings. In addition, LVEF was calculated using the Teichholz method due to the retrospective design and limited availability of archived apical views. Because this method relies on geometric assumptions, it may underestimate subtle volumetric changes compared with guideline-recommended techniques such as Simpson biplane method or 3D echocardiography. Larger prospective multicenter studies are needed to validate these findings and to clarify the clinical significance of RV–pulmonary arterial coupling alterations in anthracycline-treated patients.

## Conclusions

The present study suggests that anthracycline-induced cardiotoxicity may not be limited to LV impairment but can also involve RV function. Following anthracycline therapy, a decrease in TAPSE and the TAPSE/sPAP ratio along with an increase in pulmonary artery systolic pressure indicates a tendency toward RV systolic dysfunction. In addition, alterations in RV diastolic parameters (decrease in E/A ratio and increase in E/e′ value) suggest that both the systolic and diastolic components of RV performance may be affected.

These findings highlight the importance of incorporating RV assessment into routine evaluation of patients receiving anthracyclines. A comprehensive biventricular approach to echocardiographic monitoring may facilitate the earlier recognition of cardiotoxicity and enable timely intervention. However, given the retrospective, single-center nature and limited sample size of this study, these results should be interpreted with caution, and larger prospective studies are required to confirm these observations.

## Data Availability

The datasets supporting the conclusions of this article are included within the article. Additional data are available from the corresponding author upon reasonable request.

## Abbreviations

ASE: American Society of Echocardiography
CTRCD: Cancer therapy–related cardiac dysfunction
BSA: Body surface area
ESC: European Society of Cardiology
LV: Left ventricular
LVEDD: Left ventricular end-diastolic diameter
LVEF: Left ventricular ejection fraction
LVESD: Left ventricular end-systolic diameter
proBNP: Pro–brain natriuretic peptide
RV: Right ventricular
RVFAC: Right ventricular fractional area change
sPAP: Systolic pulmonary artery pressure
TAPSE: Tricuspid annular plane systolic excursion
